# Salmagundi

**Published:** 1886-12

**Authors:** 


### SALMAGUNDI.

EDITED BY E. G. BETTY, D.D.S.

SPEAKING of *purgatives*, Prof. Bartholow told of an old soldier who always carried about him a bullet, which he had used for *forty years* as a cathartic. It acted by its weight.—*Medical and Surgical Reporter.*

A LINIMENT FOR NEURALGIA.—Gueneau de Mussy recommends this lotion: Essence of mient, ℥ v.; tincture of aconite, ℥ ijss; chloroform, ℥ i¼. Shake thoroughly, and apply to the painful spot on a piece of flannel.—*New York Medical Journal.*

DR. MARSHALL, of Chicago, gives an account in the "Journal of the American Medical Association" for November 6th, of a superior third molar, which, after giving the patient a great deal of trouble for years, finally discharged from the posterior nares.

DR. O. W. HOLMES said in his address to the students of Edinburgh: "Literature was a good staff for walking with, but not a good one to lean upon. From Coleridge he had learned that every literary man should have some fixed and regular occupation."—*Medical and Surgical Reporter.*

A NEW journal, "The Dental Review," has made its bow to the profession—unfathered, or, as the Scotch would say, "a mitherless bairn." All the other fellows will have to mind their p's and q's now or get a good basting. The *Review* is published in Chicago, and expects to find its patronage largely in the Northwest.

---

He kissed her passionately upon her reappearance.—*Jefferson Souvenir.*

She whipped him upon his return.—*Hawkeye.*

He kissed her back.—*Constitution.*

She seated herself upon his entering.—*Albia Democrat.*

Mr. Jones walked in upon her invitation.—*Electric Light.*

We thought she sat down on being asked.—*Saturday Gossip.*

---

It may be a few days too early, but we wish all our readers a merry X-mas and a happy New Year; for, by the time our next number appears it will be too late, and so we take advantage of this opportunity. We would like to make a Christmas present to each and all, but will have to be content in trying to make Salmagundi more interesting than ever for the coming year. A helping hand all around will give variety, value, and greatly add to the general interest.

---

A very bad habit some of our medical exchanges are falling into, is placing advertisements right in the heart of the journal. Nothing is so irritating and offensive to the reader when interested in a good article as to suddenly come upon an advertisement of some cathartic pill or headache medicine. This trick may be very profitable to the publisher, but it certainly detracts from the dignity of a scientific publication, and frequently makes subscribers for other journals. We hope none of the dental journals will ever be guilty of such a misdemeanor.

---

Effects of Study on the Teeth.—Among the hard-worked pupils of the Paris public schools the teeth become deteriorated in a few weeks after entry. The second dentition is often premature. These observations confirm the statements of Dr. J. L. Williams, who has given great attention to this subject. He has shown that any mental strain shows itself upon the teeth in a short time, both in increased decay as well as in increased sensibility of dentine. Dr. D. M. Parker has reported that these same changes are always apparent in men who are training for athletic trials.—*Boston Medical Journal.*

---

DR. WILLIAM A. HAMMOND has been amusing a medical association with some humorous accounts of his experience with cocaine. He is reported to have said that there is no danger of the formation of a cocaine habit. Dr. Hughes, writing in the *Medical Review*, takes a different view, and in his summing up claims that cocaine is a tonic and stimulating exhilarant of considerable power in melancholia, mental depression, and nerve weariness, being more rapid and at the same time more evanescent in its action than morphia. He distinctly says, moreover, that, when used to excess, it intoxicates, and converts melancholia into mania, and that its continuous use is difficult to break off; that it is a dangerous therapeutic toy, and ought not to be used as a sensational plaything; that it will probably help to fill rather than to deplete the asylums, both inebriate and insane, if it should come into as general use as the other intoxicants of its class; that as an intoxicant it is more dangerous, if continuously given, than alcohol or opium, and more difficult to abandon. —*Science.*

---

A SOLVENT FOR SORDES.—Dr. A. D. MacGregor speaks highly of boric acid as a topical application in the unhealthy condition in which we frequently find the mouth, tongue, and teeth in severe cases of typhoid fever. He says, in the *British Medical Journal:* The mouth is hot; the lips dry, cracked, and glued to the sordes-covered teeth by inspissated mucus and saliva; the tongue dry, or even glazed and hard, brown or black, crusted with a fetid fur. Under these circumstances a pigment containing boric acid (30 grains), chlorate of potassium (20 grains), lemon-juice (5 fluid drachms), and glycerine (3 fluid drachms), yields comforting results. When the teeth are well rubbed with this, the sordes quickly and easily become detached; little harm will follow from the acid present. The bòric acid attacks the masses of bacilli and bacteria, the chlorate of potassium cools and soothes the mucous membrane, the glycerine and lemon-juice moisten the parts and aid the salivary secretion.—*Medical Record.*

---

ECHOES OF THE LAST ANNUAL MEETING O. S. D. S.

MICHIGAN sent about twenty to the meeting. Do it again.

"UNCLE JERRY" could not withstand the temptation, and came to the meeting with a paper that stimulated an interesting discussion.

THE voices of "Uncle Jerry" and "the other Jerry" sound so much alike that one must look twice before he is sure which one he is talking to.

THE attendance this year was the second largest in the history of the Society, almost ninety being present and participating in one way or another.

DR. TALBOT, of Chicago, interested the members very much with his method of using piano-wire springs for the correction of irregularities; a description will be found in our report.

DR. E. C. MOORE, of Detroit, Mich., was in attendance, and vowed he enjoyed it very much. We visited him at his home after the meeting, and learned several little "tricks" we shall share with our readers bye and bye.

DR. ALLPORT'S criticism on the Herbst method of filling teeth (published in another part of the REGISTER) met with a hearty reception, and provoked an animated discussion, in which it was discovered the majority agreed with him.

DR. BROPHY, of Chicago, has a matrix that certainly is a thing every dentist should have, as it materially assists in filling certain classes of cavities, though we differ with him somewhat as to the preparation of cavities and the kind of foil used.

Our friend, Dr. Jennings, was at the meeting, looking just as pretty as a peach. He had promised us that he would have a paper to read, but sickness in his family prevented his getting it ready in time. We hope to have it for the Register before a great while; he ought to make us a Christmas present of it.

Dr. Harlan's speech on the Herbst method was the effort of his life, and he did himself justice. The flash of his eye, the swelling of his chest, and the way he came down on the table with his fist, was greeted with rounds of applause time and again. Our report don't begin to give an idea, and those who staid away will never cease to regret it.

Springfield, O., is one of the liveliest and most wide awake towns of its size in the country, and we feel assured that its arrangements for the next annual meeting will be ample and thorough. President Harrison did well in appointing Dr. Runyan as Chairman of the Committee of Arrangements, for he will attend to everything in first class style.

Dr. Watt did not come among us this time, and he was sadly missed, though many thought and spoke of him. We shall see him next year, however, for Springfield is not quite an hour's ride from Xenia, and we shall then hear his familiar voice once more. He was present at the organization of the old Society in 1866, its death in 1884, and its reorganization at Chillicothe, October a year ago, and he certainly will make an effort to be at Springfield next fall.

